# Frailty increases the risk for developing urinary tract infection among 79,887 patients with diabetic mellitus and chronic kidney disease

**DOI:** 10.1186/s12877-021-02299-3

**Published:** 2021-06-07

**Authors:** Chia-Ter Chao, Szu-Ying Lee, Jui Wang, Kuo-Liong Chien, Jenq-Wen Huang

**Affiliations:** 1 Nephrology division, Department of Internal Medicine, National Taiwan University Hospital BeiHu Branch, Taipei, Taiwan; 2 Geriatric and Community Medicine Research Center, National Taiwan University Hospital BeiHu Branch, Taipei, Taiwan; 3 Graduate Institute of Toxicology, National Taiwan University College of Medicine, Taipei, Taiwan; 4 Nephrology division, Department of Internal Medicine, National Taiwan University Hospital Yunlin branch, Yunlin county, Taiwan; 5 Institute of Epidemiology and Preventive Medicine, College of Public Health, National Taiwan University, Taipei, Taiwan

**Keywords:** chronic kidney disease, diabetic kidney disease, diabetes mellitus, frail phenotype, frailty, sepsis, urinary tract infection

## Abstract

**Background:**

Patients with diabetic mellitus (DM) and chronic kidney disease (CKD) are at an increased risk of urinary tract infection (UTI) due to their altered immunological integrity. These patients are similarly prone to developing frailty, a state of cumulative health deficits involving multiple domains and leading to adverse outcomes. Whether frailty predisposes affected individuals to UTI among patients with DM and CKD remains unclear.

**Methods:**

A population-based cohort of patients with DM and CKD (*n* = 79,887) were assembled from the Longitudinal Cohort of Diabetes Patients, with their baseline frailty status measured by a  modified FRAIL scale. We analyzed their risk of developing UTI depending on their severity of frailty, after accounting demographic profiles, lifestyle factors, comorbidities, concurrent medications, and major interventions. A secondary analysis focused on the risk of urosepsis related to frailty.

**Results:**

Among all participants, 36.1 %, 50.3 %, 12.8 %, and 0.8 % did not have or had 1, 2, and ≥ 3 FRAIL items, respectively, at baseline. After 3.51 years, 11,175 UTI events occurred. Kaplan-Meier analysis showed that participants with DM, CKD and an increasing number of FRAIL items had successively higher incidence of UTI than those without any FRAIL items (log rank *p* < 0.001). Cox proportional hazard modeling revealed that after accounting for all confounders, those with more severe frailty exhibited a significantly higher risk of incident UTI (for groups of 1, 2, and ≥ 3 FRAIL items, hazard ratio 1.19, 1.24, and 1.43, respectively; all *p* < 0.001) than those without. An 11 % risk elevation for UTI could be observed for every FRAIL item increase. Participants with more severe frailty exhibited a trend of having higher risk of urosepsis as well.

**Conclusions:**

Having frailty predicted a higher risk of developing UTI in the future in patients with DM and CKD. It would be prudent to screen for frailty in these patients and provide optimal frailty-directed management to attenuate their risk of UTI and improve their outcomes.

## Introduction

The number of patients living with diabetes mellitus (DM) are increasing globally. According to the diabetes atlas, one-fifth of older adults worldwide have DM [1], and DM accounts for 11.3 % of global mortality [2]. Complications occur in one-fourth to half patients with DM especially microvascular ones, among which diabetic kidney disease (DKD), defined according to the status of a decreased estimated glomerular filtration rate (eGFR) level and micro- to overt- albuminuria [3], accounts for a substantial proportion [4]. Roughly 10 % mortality cases with DM can be attributable to a renal origin, and DKD causes nearly 50 % cases of end-stage renal disease (ESRD) [5]. As the population with DM and CKD are still expanding, we should pay more attention to the management of complications resulting from the presence of DM and CKD.

Immune system can be compromised in patients with DM and CKD. Patients with DM exhibit a 1.2- to 2-fold higher risk of infection and related hospitalization compared to those without [6], and those with poorly controlled DM are at more than 3-fold higher risk of acquiring any infections, including wound, airway or urinary tract infection (UTI) than those with well controlled DM [7]. Intensive glycemic control can substantially curb the risk of infectious complications [8]. UTI is a common and outcome-modifying type of infections among patients with diabetes [6, 9]; a population-based study reported that 8.2 % diabetic patients developed UTI annually, while one-third of them had prior experiences of UTI [10]. The development of UTI leads to higher out-patient medical costs [9], supporting the economic importance of UTI in diabetic patients. In addition, the presence of chronic kidney disease (CKD) also impairs immunity; innate and adaptive immunity are both aberrantly regulated in patients with CKD [11], while cumulative uremic toxin exposures further lead to chronic inflammation, multimorbidity, and immunocyte dysfunction. Consequently, patients with DM and CKD are undoubtedly at a greater risk of developing infection, particularly UTI, compared to those without.

Categories of risk factors for UTI in patients with DM and CKD include genetic susceptibility, impairment in host defense machineries and/or defective urinary tract anatomy [12]. CKD deteriorates host immunity and alters host responses to invading microorganisms; changes in the composition of urine such as hypercalciuria and glycosuria, or congenital anomaly involving the urinary tract all increase the risk of UTI [12]. Importantly, prior experiences of UTI in an individual significantly raise the risk of subsequent UTI, a phenomenon suggesting that certain clinical features can be responsible for one’s susceptibility to UTI. Frailty, a term utilized to describe older adults’ susceptibility to adverse outcomes stemming from cumulative subclinical deficits in multiple dimensions [13], bears much resemblance to the contextual property of this host feature. None of the existing studies addresses the relationship between frailty and the risk of UTI in a given population. This consideration prompted us to hypothesize that having frailty might place patients with DM and CKD at an even higher risk of UTI compared to those with DM and CKD but without frailty. We attempted to answer this question using a well characterized population-based cohort of patients with DM.

## Methods

### Identifying and grouping participants

**Fig. 1:**
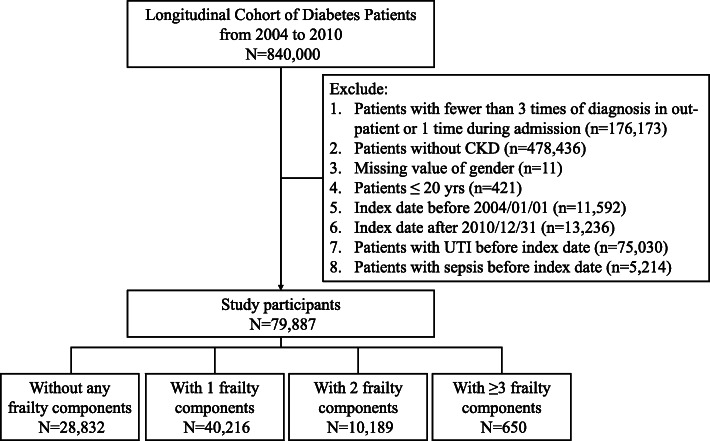
The flow chart of participant assembly in the current study. *CKD, chronic kidney disease; UTI, urinary tract infection*

In this study, the Longitudinal Cohort of Diabetes Patients (LCDP) was harnessed for participant identification and analysis. LCDP is a well-maintained cohort of Taiwanese patients (n = 840,000) with at least one time of DM diagnosis during the period between 2004 and 2010, and has generated multiple publications related to the epidemiology of diabetic complications [14–16]. To strengthen the diagnosis of DM, we first restricted participants to those with ≥ 3 out-patient or ≥ 1 in-patient diagnosis of diabetes (Fig. 1). Among these patients, we further identified those with DM and CKD by imposing the requirement of acquiring CKD after the onset of DM, a strategy we adopted previously [15]. Briefly, we identified patients with CKD based on a validated set of diagnostic codes applicable for use in registry studies, to which our cohort belonged as well. The complete set of codes has been published previously in our prior studies [14, 15, 17]. According to a validation study using Taiwan National Health Insurance Research Database (NHIRD), this diagnostic code combination had an 81.8 % sensitivity and 99.3 % specificity for diagnosing CKD, while the positive and negative predictive values for diagnosing CKD were 87.3 and 99.0 %, respectively [18]. The content of these diagnostic codes captures an extensive range of structural or functional kidney disorders, and is not narrowly focused on renal function decline only. This approach complies with the recent refinement of the concept of CKD proposed by the Kidney Disease Improving Global Outcomes (KDIGO) [19]. Index date was defined as the day when patients met the definition of both DM and CKD. Exclusion criteria comprised of those with missing data, pediatric patients, those with an index date prior to January 1st, 2004 or after December 31st, 2010 to permit an adequate length of follow up (at least 1 year), and those who developed the outcomes of interest (UTI and urosepsis) in this study (Fig. 1). After applying the exclusion criteria, participants were divided into those without and with different levels of frailty based on the criteria outlined below. We collected all clinical features at the beginning of their follow-up. The severity of DM was gauged by the adapted diabetes complications severity index (aDCSI) [20]. We followed the enrollees from the index date to the end of follow-up (December 31st, 2011), the development of outcomes, or when they died, whichever occurred first.

### Participant stratification scheme

Participants were divided into groups according to the results of the FRAIL scale. FRAIL scale is a convenient frailty-screening tool originally designed to rapidly identify middle aged-to-older individuals with an impaired activity of daily living, poor physical performance, and an increased risk of mortality [21]. FRAIL scale has been tested and validated in multiple different populations besides its founding one, including patents with renal failure [22], lung diseases [23], and institutionalized ones [24]. The performance of FRAIL scale has been compared to those of other frailty-classification schemes such as Cardiovascular Health Study (CHS) and Study of Osteoporotic Fractures (SOF) scales [25], with a superior outcome-predictive efficacy reported for FRAIL scale. A recent international practice guideline endorsed the use of FRAIL scale as an optimal screening instrument for detecting frailty [26].

FRAIL scale measures deficit accumulation in 5 domains (or items), namely fatigue, resistance, ambulation, illnesses, and loss of body weight [21]. Individuals with more numbers of FRAIL items exhibit higher frail severity, with a dose-responsive relationship [14]. We operationalized each item of the FRAIL scale using diagnostic code combinations, the constituents of which were selected based on keyword research, literature review, and expert review followed by several rounds of discussions and consensus reached by in-house geriatricians and epidemiologists [14]. The Fatigue item was substituted by diagnoses containing any of the following keywords (fatigue, malaise, asthenia, weakness, etc.). The Resistance item originally involved the report of perceived difficulty in stair climbing, and we mobilized this item using diagnoses pertaining to fall with/without related injuries and debility. The Ambulation item assessed the inability of walking, and was operationalized using diagnostic codes whose content involved walking difficulty and gait abnormalities. We kept the original content of the Illness item by identifying those with ≥ 4 out of the same 10 morbidities originally specified in the FRAIL scale (excluding diabetes). The Loss of weight item was mobilized using diagnoses containing relevant keywords (malnutrition, muscle wasting, feeding difficulty, cachexia, etc.). The complete list of codes can be found in our published work previously [14]. Patients were deemed positive for individual item if they had any of the diagnostic code combinations during ≥ 2 out-patient clinics or ≥ 1 hospitalization episode within the preceding years of the index date. The utilization of this approach has been found to be quite informative in identifying patients with more physically prominent frailty, as patients with frailty based on this modified FRAIL scale have a significantly higher risk of delirium, adverse outcomes, and higher healthcare utilization than those without [14, 15, 27, 28]. Participants were categorized based on whether they did not have, or had 1, 2, and ≥ 3 FRAIL items, with regard to the risk of developing the outcomes of this study.

### Outcome definition

The primary outcome of interest in this study was the development of UTI. We identified UTI events during follow-up based on the International Classification of Diseases, 9th revision, Clinical Modification (ICD-9-CM) codes 590.x (excluding 590.0), 595.0, 595.3, 595.4, 595.8, 595.9, 596.81, 597.x, 599.0, and 601.x. These codes were derived from prior studies attempting to capture all UTI-related out-patient visits and hospitalizations [29, 30]. The quality of using these ICD-9-CM codes to identify UTI cases was found to be excellent, with 85–93 % positive predictive value (PPV) [29], while another validation study similarly reported that ICD codes accurately identified those with UTI in the primary care setting (PPV 81–88 %) [31]. The secondary endpoint was the development of urosepsis, a more severe complication of UTI, defined by the combination of UTI and sepsis diagnoses. The diagnostic codes of sepsis were also derived from existing reports [32, 33].

### Statistical analysis

We used means with standard deviations (SDs) and numbers with percentages in parentheses for describing continuous and categorical variables, respectively. Data between participants without and with different numbers of FRAIL items were compared using one-way analysis of variance (ANOVA). First, we compared the demographic profiles (age and gender), lifestyle factors (smoking and alcoholism), obesity, comorbidities (including Charlson comorbidity index [CCI] [34], the severity of DM [aDCSI], concurrent medications, and major interventions within the preceding year between each group. After follow-up, we calculated the UTI event counts and incidence density for each group. Kaplan-Meier survival analysis was employed to construct cumulative event curves for each group, followed by comparisons using the log-rank test. We then conducted Cox proportional hazard regression to analyze the risk of developing UTI, incorporating demographic profiles, lifestyle factors, obesity, comorbidities, medications, major interventions, and FRAIL item counts. Another set of Cox proportional hazard regression was done using the development of urosepsis as the dependent variable, incorporating the same set of variables. We performed subgroup analyses based on different age strata (< 65 and ≥ 65 years), gender, and renal function categories. A competing risk analysis incorporating mortality as the competing event was also performed. A sensitivity analysis incorporating the same set of variables and individual FRAIL items was also performed. In all analyses, a *p* value < 0.05 was considered statistically significant.

### Ethical statement

This study has been approved as a subpart of a large project by the Institutional Review Board of National Taiwan University Hospital (NO. 201802063W). Informed consent was deemed unnecessary by the review board due to data anonymization and participant identity scrambling prior to participant identification. The protocol of this study adhered to the Declaration of Helsinki.

## Results

From the LCDP during the study period, we identified 79,887 patients with DM and CKD after applying the exclusion criteria (Fig. 1). These participants were divided into those without any FRAIL item (*n* = 28,832, 36.1 %), with 1 (*n* = 40,216, 50.3 %), 2 (*n* = 10,189, 12.8 %), and ≥ 3 (*n* = 650, 0.8 %) FRAIL items, respectively, at the beginning of this study. Those with increasing numbers of FRAIL items, or a higher frail severity, were significantly older, more likely to smoke or have alcoholism, had a higher prevalence of most comorbidities (cardiovascular, metabolic, pulmonary, hepatobiliary, oncologic, neuropsychiatric, and orthopedic ones) and were more likely to receive certain types of medications compared to those without any FRAIL item (Table 1). Participants with DM, and CKD and more FRAIL items had significantly greater severity of DM than those with DM, CKD but without any FRAIL item.

**Table 1:** Features of participants with diabetic kidney disease without and with different severities of frailty

	Total (*n* = 79,887)	No FRAIL item (*n* = 28,832)	1 FRAIL item (*n* = 40,216)	2 FRAIL items (*n* = 10,189)	≥ 3 FRAIL items (*n* = 650)	p-value*
*Baseline clinical profiles*						
**Age (years)**	59.6 ± 14.0	52.5 ± 12.4	62.9 ± 13.3	65.9 ± 12.9	69.9 ± 12.5	*< 0.001*
**Sex (Female %)**	24,240 (30.3)	7,528 (26.1)	12,939 (32.2)	3,560 (34.9)	213 (32.8)	*< 0.001*
**Obesity (%)**	1,397 (1.8)	496 (1.7)	727 (1.8)	169 (1.7)	5 (0.8)	*0.167*
**Alcoholism (%)**	1,161 (1.5)	435 (1.5)	540 (1.3)	166 (1.6)	20 (3.1)	*< 0.001*
**Smoking (%)**	667 (0.8)	208 (0.7)	334 (0.8)	115 (1.1)	10 (1.5)	*< 0.001*
*Diabetic severity*^a^	0.8 ± 1.2	0.5 ± 0.9	1.0 ± 1.3	1.2 ± 1.4	1.5 ± 1.4	*< 0.001*
*Morbidity profile*						
**Hypertension (%)**	55,620 (69.6)	11,667 (40.5)	34,242 (85.2)	9,129 (89.6)	582 (89.5)	*< 0.001*
**Hyperlipidemia (%)**	43,179 (54.1)	14,205 (49.3)	22,589 (56.2)	6,029 (59.2)	356 (54.8)	*< 0.001*
**ACS (%)**	21,019 (26.3)	2,411 (8.4)	13,927 (34.6)	4,356 (42.8)	325 (50.0)	*< 0.001*
**Atrial fibrillation (%)**	10,544 (13.2)	1,271 (4.4)	6,765 (16.8)	2,328 (22.9)	180 (27.7)	*< 0.001*
**PVD (%)**	2,208 (2.8)	393 (1.4)	1,264 (3.1)	516 (5.1)	35 (5.4)	*< 0.001*
**Cerebrovascular disease (%)**	14,225 (17.8)	294 (1.0)	10,372 (25.8)	3,266 (32.1)	293 (45.1)	*< 0.001*
**Heart failure (%)**	8,072 (10.1)	161 (0.6)	5,979 (14.9)	1,792 (17.6)	140 (21.5)	*< 0.001*
**COPD (%)**	11,831 (14.8)	410 (1.4)	8,238 (20.5)	2,930 (28.8)	253 (38.9)	*< 0.001*
**Malignancy (%)**	6,528 (8.2)	685 (2.4)	4,385 (10.9)	1,347 (13.2)	111 (17.1)	*< 0.001*
**Chronic liver disease (%)**	30,130 (37.7)	9,684 (33.6)	15,229 (37.9)	4,888 (48.0)	329 (50.6)	*< 0.001*
**Stage 5 CKD (%)**	1,401 (1.8)	350 (1.2)	838 (2.1)	203 (2.0)	10 (1.5)	*< 0.001*
**Parkinsonism (%)**	1,239 (1.6)	99 (0.3)	748 (1.9)	337 (3.3)	55 (8.5)	*< 0.001*
**Mental disorders (%)**	13,792 (17.3)	2,669 (9.3)	7,809 (19.4)	3,059 (30.0)	255 (39.2)	*< 0.001*
**Osteoarthritis (any site) (%)**	25,665 (32.1)	3,952 (13.7)	15,897 (39.5)	5,384 (52.8)	432 (66.5)	*< 0.001*
**Gout (%)**	24,840 (31.1)	6,146 (21.3)	14,212 (35.3)	4,201 (41.2)	281 (43.2)	*< 0.001*
**Hypoglycemia events (%)**	204 (0.3)	37 (0.1)	109 (0.3)	52 (0.5)	5 (0.8)	*< 0.001*
*CCI*	*3.3 ± 2.1*	*2.4 ± 1.4*	*3.7 ± 2.1*	*4.3 ± 2.3*	*5.1 ± 2.5*	*< 0.001*
*Concomitant medications*						
**ACEi (%)**	27,459 (34.4)	8,596 (29.8)	14,888 (37.0)	3,751 (36.8)	224 (34.5)	*< 0.001*
**Allopurinol (%)**	5,090 (6.4)	1,216 (4.2)	3,013 (7.5)	805 (7.9)	56 (8.6)	*< 0.001*
**Anti-depressants (%)**	14,294 (17.9)	3,852 (13.4)	7,792 (19.4)	2,493 (24.5)	157 (24.2)	*< 0.001*
**Anti-psychotics (%)**	17,513 (21.9)	4,972 (17.2)	9,331 (23.2)	2,987 (29.3)	223 (34.3)	*< 0.001*
**ARB (%)**	38,331 (48.0)	11,298 (39.2)	21,549 (53.6)	5,196 (51.0)	288 (44.3)	*< 0.001*
**Aspirin (%)**	31,476 (39.4)	7,866 (27.3)	18,473 (45.9)	4,843 (47.5)	294 (45.2)	*< 0.001*
**Benzodiazepine (%)**	37,876 (47.4)	11,004 (38.2)	20,499 (51.0)	5,997 (58.9)	376 (57.9)	*< 0.001*
**β-blockers (%)**	36,936 (46.2)	10,138 (35.2)	21,113 (52.5)	5,368 (52.7)	317 (48.8)	*< 0.001*
**Clopidogrel (%)**	5,905 (7.4)	1,029 (3.6)	3,852 (9.6)	968 (9.5)	56 (8.6)	*< 0.001*
**COX-II inhibitor (%)**	20,262 (25.4)	4,224 (14.7)	12,094 (30.1)	3,683 (36.2)	261 (40.2)	*< 0.001*
**Fibrate (%)**	16,717 (20.9)	6,431 (22.3)	8,209 (20.4)	1,979 (19.4)	98 (15.1)	*< 0.001*
**NSAID (%)**	70,560 (88.3)	25,140 (87.2)	35,772 (89.0)	9,096 (89.3)	552 (84.9)	*< 0.001*
**Statin (%)**	36,345 (45.5)	13,115 (45.5)	18,677 (46.4)	4,328 (42.5)	225 (34.6)	*< 0.001*
**Warfarin (%)**	2,172 (2.7)	335 (1.2)	1,465 (3.6)	349 (3.4)	23 (3.5)	*< 0.001*
*Anti-diabetic medications*						
**α-glucosidase inhibitor (%)**	12,937 (16.2)	5,186 (18.0)	6,298 (15.7)	1,391 (13.7)	62 (9.5)	*< 0.001*
**Biguanide (%)**	44,897 (56.2)	18,501 (64.2)	21,211 (52.7)	4,945 (48.5)	240 (36.9)	*< 0.001*
**DPP4 inhibitors (%)**	8,324 (10.4)	3,753 (13.0)	3,784 (9.4)	755 (7.4)	32 (4.9)	*< 0.001*
**Insulin (%)**	9,990 (12.5)	4,152 (14.4)	4,665 (11.6)	1,110 (10.9)	63 (9.7)	*< 0.001*
**Meglitinide (%)**	9,966 (12.5)	3,815 (13.2)	4,967 (12.4)	1,121 (11.0)	63 (9.7)	*< 0.001*
**Sulfonylurea (%)**	42,621 (53.4)	17,628 (61.1)	20,123 (50.0)	4,646 (45.6)	224 (34.5)	*< 0.001*
**Thiazolidinedione (%)**	9,249 (11.6)	4,408 (15.3)	4,007 (10.0)	796 (7.8)	38 (5.9)	*< 0.001*
*Major treatment procedures within 1 year*						
**Coronary revascularization (%)**	1,260 (1.6)	104 (0.4)	943 (2.3)	204 (2.0)	9 (1.4)	*< 0.001*
**Cardiac surgery (any) (%)**	2,116 (2.7)	161 (0.6)	1,562 (3.9)	372 (3.7)	21 (3.2)	*< 0.001*
*Any hospitalization (%)*	29,218 (36.6)	7,779 (27.0)	16,433 (40.9)	4,644 (45.6)	362 (55.7)	*< 0.001*

*ACEi* angiotensin-converting enzyme inhibitor, *ACS* acute coronary syndrome, *ARB* angiotensin receptor blocker, *CCI* Charlson comorbidity index, *CKD* chronic kidney disease, *COPD* chronic obstructive pulmonary disease, *COX* cyclo-oxygenase, *DPP4* dipeptidyl peptidase 4, *NSAID* non-steroidal anti-inflammatory drug, *PVD* peripheral vascular disease

* Compared between 0, 1, 2, and ≥ 3 items groups, using the analysis of variance (ANOVA)

^a^ Based on the adapted diabetes complications severity index (aDCSI)

**Fig. 2:**
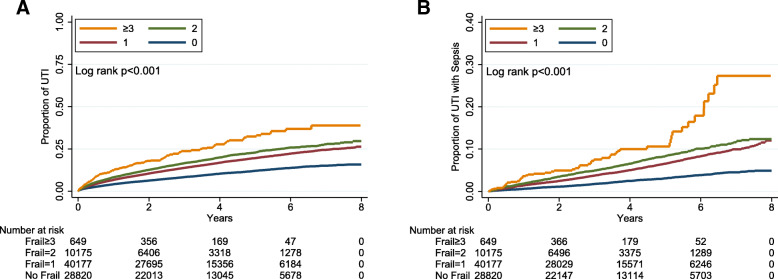
Kaplan-Meier cumulative event curves for (**A**) urinary tract infection and (**B**) urosepsis, according to the number of FRAIL item counts. *UTI, urinary tract infection*

Among those with at least 1 FRAIL item, Illness was the most prevalent out of the 5 items (58 %), followed by Fatigue (17.1 %) and Loss of weight (1.3 %) (Table 2). After a mean 3.51 years of follow-up, totally 11,175 UTI events occurred, equivalent to an incidence density of 39.8 events per 1000 person-year. Kaplan-Meier event curve analysis showed that participants with DM and CKD and an increasing numbers of FRAIL items had a progressively higher incidence of UTI than those without any FRAIL item (log rank *p* < 0.001) (Fig. 2 A). Univariate analysis found that participants with DM and CKD and 1, 2, and ≥ 3 FRAIL items had a hazard ratio (HR) of 1.7 (95 % confidence interval [CI] 1.63–1.77), 2.03 (95 % CI 1.92–2.16), and 3.03 (95 % CI 2.57–3.58) for developing UTI than those without any items (Table 3). Cox proportional hazard modeling revealed that after accounting for all variables in Table 1, having more severe frailty was predictive of a higher risk of UTI (for groups of 1, 2, and ≥ 3 FRAIL items, HR 1.19, 1.24, and 1.43, respectively; all *p* < 0.001) (Table 3). An average of 11 % risk elevation for UTI was observed for every FRAIL item increase. After accounting for mortality as the competing event, having frailty at the beginning of follow-up was still associated with a significantly higher risk of UTI during follow up, with a dose-response relationship (for 1, 2, and > 2 FRAIL items, HR 1.14, 1.16, and 1.28, 95 % CI 1.08–1.21, 1.08–1.25, and 1.06–1.53, respectively) (Table 3). We found an essentially similar dose-responsive relationship between the severity of frailty and the risk of developing UTI in patients with < 65 and ≥ 65 years, male and female patients, and those with earlier stages of CKD (Table 4). However, the estimation of risk did not reach significance in those with stage 5 CKD due to a low patient number in that subcohort.

**Table 2:** The item distribution of FRAIL scale depending upon the severities of frailty

	Total (*n* = 79,887)	No FRAIL item (*n* = 28,832)	1 FRAIL item (*n* = 40,216)	2 FRAIL items (*n* = 10,189)	≥ 3 FRAIL items (*n* = 650)
*Component*					
**Fatigue (%)**	13,685 (17.1)	0 (0)	4,111 (10.2)	8,959 (87.9)	615 (94.6)
**Resistance (%)**	1,050 (1.3)	0 (0)	190 (0.5)	547 (5.4)	313 (48.2)
**Ambulation (%)**	474 (0.6)	0 (0)	48 (0.1)	295 (2.9)	131 (20.2)
**Illness (%)**	46,297 (58.0)	0 (0)	35,620 (88.6)	10,030 (98.4)	647 (99.5)
**Loss of weight (%)**	1,072 (1.3)	0 (0)	247 (0.6)	547 (5.4)	278 (42.8)

**Table 3:** Risk of developing urinary tract infection and urosepsis according to the severity of frailty of participants

Outcomes	Events	Person-year	Incidence density*	Crude		Model A^&^		Model B^#^	
				**HR**	**95 % CI**	**HR**	**95 % CI**	**HR**	**95 % CI**
*Urinary tract infection*									
No FRAIL item	2,934	110,373.7	26.6	1	-	1	-	1	-
1 item	6,314	137,015.7	46.1	1.70	1.63–1.77^a^	1.19	1.12–1.26^a^	1.14	1.08–1.21^a^
2 items	1,778	31,590.2	56.3	2.03	1.92–2.16^a^	1.24	1.16–1.34^a^	1.16	1.08–1.25^a^
≥ 3 items	149	1,723.1	86.5	3.03	2.57–3.58^a^	1.43	1.21–1.70^a^	1.28	1.06–1.53^b^
*Per 1 item increase*				*1.45*	*1.41–1.49*^*a*^	*1.11*	*1.08–1.15*^*a*^	*1.07*	*1.04–1.11*^*a*^
*Urinary tract infection with sepsis*									
No FRAIL item	699	110,869.5	6.3	1	-	1	-	1	-
1 item	1,910	138,441.3	13.8	2.20	2.02–2.40^a^	1.07	0.95–1.19	1.004	0.90–1.12
2 items	559	31,917.7	17.5	2.80	2.51–3.13^a^	1.13	0.98–1.29	1.005	0.88–1.15
≥ 3 items	53	1782.0	29.7	4.79	3.62–6.33^a^	1.18	0.88–1.59	1.048	0.77–1.42
*Per 1 item increase*				*1.68*	*1.60–1.76*^*a*^	*1.06*	*0.995–1.13*	*1.008*	*0.95–1.07*

^*^ per 1000 patient-year

^&^ Incorporating all variables in Table 1

^#^ Model A with competing risk analysis for mortality

^*a*^ *p < 0.001*

^*b*^ *p < 0.01*

**Table 4:** Risk of developing urinary tract infection according to different subgroups

**Outcomes**	**Age < 65 years** **(*n* = 51,071)**		**Age ≥ 65 years** **(*n* = 28,816)**	
	**HR**	**95 % CI**	**HR**	**95 % CI**
No FRAIL item	1	–	1	–
1 item	1.16	1.08–1.24^a^	1.17	1.06–1.28^b^
2 items	1.23	1.11–1.37^b^	1.22	1.10–1.36^b^
≥ 3 items	1.05	0.71–1.54	1.54	1.25–1.88^a^
*Per 1 item increase*	*1.11*	*1.06–1.16*^*a*^	*1.10*	*1.05–1.15*^*a*^
		**Female** **(*n* = 24,240)**	**Male** **(*n* = 55,647)**	
	**HR**	**95 % CI**	**HR**	**95 % CI**
No FRAIL item	1	–	1	–
1 item	1.21	1.11–1.31^a^	1.17	1.09–1.26^a^
2 items	1.31	1.18–1.46^a^	1.19	1.08–1.31^b^
≥ 3 items	1.51	1.14–1.99^b^	1.32	1.06–1.65^c^
*Per 1 item increase*	*1.14*	*1.08–1.20*^*a*^	*1.09*	*1.04–1.14*^*b*^
	**Earlier stage CKD** **(*n* = 78,486)**		**Stage 5 CKD** **(*n* = 1,401)**	
	**HR**	**95 % CI**	**HR**	**95 % CI**
No FRAIL item	1	–	1	–
1 item	1.19	1.13–1.26^a^	1.17	0.76–1.78
2 items	1.24	1.16–1.34^a^	1.28	0.73–2.25
≥ 3 items	1.42	1.19–1.69^a^	2.15	0.57–8.07
*Per 1 item increase*	*1.11*	*1.07–1.15*^*a*^	*1.16*	*0.89–1.51*

^*a*^ *p < 0.001*

^*b*^ *p < 0.01*

^*c*^ *p < 0.05*

A secondary analysis focused on the risk of developing urosepsis among participants with DM and CKD according to the severity of frailty. The incidence density of urosepsis in these patients was 11.4 events per 1000 person-year. Kaplan-Meier analysis similarly showed that participants with DM, CKD, and an increasing number of FRAIL items had rising incidence of urosepsis (log rank *p* < 0.001; Fig. 2B) and a higher risk over time than those without any FRAIL item (Table 3). Cox proportional hazard regression showed that there was a trend of increasing urosepsis risk when participants had a higher frail severity among patients with DM and CKD (Table 3).

A sensitivity analysis incorporating individual FRAIL item positivity instead of FRAIL item counts was performed (Table 5). We found that positivity involving the Fatigue (HR 1.06, 95 % CI 1.01–1.12) or Illness (HR 1.22, 95 % CI 1.15–1.29) item was independently associated with a higher risk of developing UTI among patients with DM and CKD. In addition, positivity involving the Ambulation item (HR 1.47, 95 % CI 1.08–1.99) was independently associated with a higher risk of developing urosepsis (Table 5).

**Table 5:** Risk of developing urinary tract infection and urosepsis according to individual FRAIL item positivity

Outcomes	Crude		Model A^&^	
	**HR**	**95 % CI**	**HR**	**95 % CI**
*Urinary tract infection*				
Fatigue	1.21	1.16–1.27^a^	1.06	1.01–1.12^c^
Resistance	1.30	1.12–1.51^b^	0.998	0.86–1.16
Ambulation	1.91	1.57–2.32^a^	1.17	0.97–1.43
Illness	1.81	1.74–1.88^a^	1.22	1.15–1.29^a^
Loss of weight	1.83	1.60–2.09^a^	1.02	0.89–1.16
*Urinary tract infection with sepsis*				
Fatigue	1.21	1.11–1.33^a^	1.35	0.95–1.14
Resistance	1.49	1.15–1.94^b^	1.03	0.79–1.34
Ambulation	2.93	2.17–3.95^a^	1.47	1.08–1.99^c^
Illness	2.43	2.25–2.63^a^	1.07	0.96–1.20
Loss of weight	2.82	2.29–3.46^a^	1.04	0.84–1.28

^&^ Incorporating all variables in Table 1

^*a*^ *p < 0.001*

^*b*^ *p < 0.01*

^*c*^ *p < 0.05*

## Discussion

In the current study, we identified and assembled a large group of patients with DM and CKD, followed by characterizing their baseline frailty status and subsequent follow-up. We showed for the first time that having frailty could predict their risk of developing UTI and a tendency for more severe episodes during the years to come in these patients. The relationship between FRAIL scale results and the risk of UTI was not mediated solely by the Illness item, which was the most prevalent one. Judging from the fact that UTI aggravates renal outcomes in patients with CKD [35] and consumes excessive healthcare resources in patients with DM and CKD [9], it would be prudent to screen for frailty in these patients and provide them with optimal frailty-directed management in order to attenuate their risk of UTI and improve their outcomes.

**Fig. 3:**
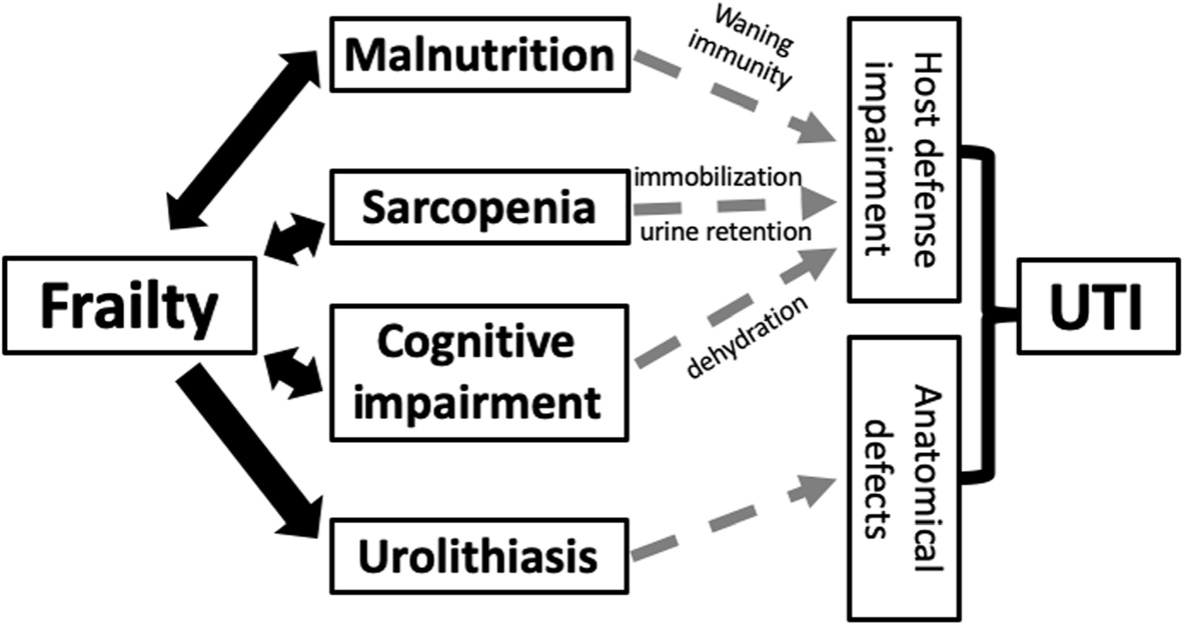
A diagram outlining the potential mechanisms through which frailty may predispose one to an increased risk of urinary tract infection. *UTI, urinary tract infection*

Uncontrolled hyperglycemia can impair the function of polymorphonuclear cells through decreasing their mobility and chemotactic ability, as well as compromise the performance of other immunocytes including macrophages and lymphocytes [36]. Complement dysfunction and defective humoral immunity also play a role in the relatively immune-suppressive status conferred by DM and CKD [37]. On the other hand, the association between frailty and the risk of UTI has not been addressed before, but preliminary evidence from other population supports the possibility of such relationship. A recent large cohort study revealed that frailty prior to the receipt of immunosuppressive therapies significantly elevated the risk of infection in patients with inflammatory bowel disease [38]. This risk surge was independent of age, other morbidities, and medications. Another systematic review summarized that the results of frail index were able to predict the risk of contracting healthcare associated infections among medically and surgically treated patients [39]. Our findings further extend their observations that frailty begets infection, by showing that frailty confers an undue risk for a specific infection type among those who are particularly susceptible. Several reasons may be responsible for the observed relationship between frailty and the UTI risk. Patients with frailty frequently have co-existing malnutrition and other geriatric syndromes including sarcopenia, cognitive dysfunction, and/or depression [40, 41]. Prior studies showed that having low mini-nutrition assessment (MNA) scores was associated with an increased UTI risk [42]. Inadequate dietary intake may precipitate hypovitaminosis D, a potential risk factor for UTI in different populations [43]. Older adults with cognitive dysfunction, especially those who have dementia, are more likely to develop dehydration, accidental falls, and UTI compared to those without [44]. Sarcopenia frequently accompanies frailty and correlates with an increased likelihood of urine retention, catheterization and impaired mobility, all of which contribute to the risk of UTI among vulnerable population such as in-patients [45]. Finally, a recent study discovered that having frailty placed an individual at a higher risk of developing urolithiasis through multiple mechanisms including hypercalciuria and immobilization [27], and UTI can be a potential complication resulting from structural damages caused by urolithiasis. We thus provided a putative summary of the potential mechanisms responsible for the observed relationship between frailty and the UTI risk based on the existing literature in Fig. 3.

Although we discovered that frailty increased the risk of UTI in patients with DM and CKD, the association between frailty and urosepsis exhibited a dose-responsive trend only (Table 3). It is possible that the severity of frailty we identified in this cohort may differ from results using other screening instruments, since FRAIL scale has a strong flooring effect and identifies fewer patients with frailty but with higher specificity [46]. A lower degree of frailty among the categorized participants may dilute the risk of developing a more severe outcome such as urosepsis relative to UTI among the entire group of participants. In addition, the relatively lower incidence of outcome (urosepsis) may also account for the paucity of a significant association between frailty and the urosepsis risk. Indeed, a population-based registry analysis from the United Kingdom involving nearly 4 million primary care patients showed that having frailty was associated with a significantly higher risk of sepsis, in which UTI constituted the main origin [47]. We expect that expanding the size of our cohort in a future study would facilitate the detection of this association.

Phenotyping the risk factors of UTI can be important both in terms of diagnosis/treatment and prognostication, based on the European Association of Urology recommendations [12]. Since frailty may alter the risk of UTI and potentially aggravate the outcomes of those with infections, we suggest that frailty could serve as a type “E” risk factor for UTI, meaning that frailty is an extra-urogenital risk factor and worsens outcome at the same time. In this sense, strategies aiming to ameliorate frailty may favorably alter the risk of UTI in patients with DM and CKD. There are several promising approaches with potential benefits of preventing frailty onset or slowing its progression, including the enhancement of physical activity, optimization of proteinaceous food intake, avoiding potentially inappropriate medication prescription, alone or in combination as an integrated program. However, in patients with DM and CKD, nutritional intervention needs to be cautious, since overzealous protein supplementation aiming to correct frailty may take the toll of more severe metabolic disturbance and deteriorate renal function. Patients with DM and CKD are also at risk of vitamin D insufficiency, which has been shown to modify the risk of frailty [48] and serves as a potential manageable target. Judging from these findings, it is prudent to devise an individualized frailty-oriented care plan tailored to the health status of patients with DM and CKD, in order to attenuate their risk of UTI. Nonetheless, more studies are needed to support the utility of this risk-mitigation strategy.

Several medications were used less frequently in patients with higher FRAIL item counts, including angiotensin-converting enzyme inhibitors, angiotensin II receptor blockers, β-blockers, clopidogrel, fibrate, and statin, compared to those without any FRAIL item (Table 1). Indeed, treatment intensity for multiple illnesses such as malignancies [49], hypertension [50], or the accessibility to medical specialist care [51] has been found to be lower among frail patients than that among non-frail ones, despite the potentially higher prevalence of these morbidities in the former group. There are also calls for safely de-prescribing relevant medications among frail older adults to avoid polypharmacy and the associated adverse sequels [50]. This is not equivalent to under-treatment for these frail patients, since their physical condition may not allow such intensive treatments. Nonetheless, we have accounted for all variables, including the morbidity status and the associated medications, in our regression analyses. Consequently, we believe that imbalances in clinical features between groups may not influence the overall validity of our findings.

Our study has its strengths and weakness. This idea has not been tested before in the literature and our results prominently enrich the existing literature. The large number of our cohort, the extensive variables considered during analyses, and a sufficient follow-up duration all increase the validity of our findings. The strategy of defining the exposure (frailty) and outcome (UTI) variable in this study has been adopted in the existing literature [14, 15, 31, 33], with credible results obtained. However, several features of this study warrant consideration before cautiously interpreting our findings. We did not arrange image examinations for urinary tract anatomy among participants and thus could not delineate the specific structural factors through which frailty might increase the risk of UTI in these patients. Second, laboratory parameters were unavailable in our cohort as we relied on diagnostic codes for identifying events. We were unable to adjust for data such as pyuria severity, leukocyte counts, or inflammatory indices in our analyses. Nonetheless, our participants were identified from the entire population with sufficiently large numbers, and we believe that the representativeness of these patients could negate most potential imbalances in laboratory data. We did not have information about these patients’ past history of UTI, so this variable was not included in our analyses. Finally, code-based identification of frailty could have intrinsic limitations due to the possibility of under-coding and variations in patient characteristics. Those who were diagnosed with specific morbidities and coded in the database tend to have more severe illnesses. A prior study comparing the agreement between administrative database and hospital records showed a 63–70 % false negative rate for certain comorbidities [52], suggesting that under-coding might not be uncommon. Thus, it is plausible that over-estimation of effect size could occur. However, this approach of using administrative database to identify frailty can still be advantageous if a large cohort is needed for testing study hypothesis. In addition, the demonstration of a dose-responsive relationship between frailty and its effect on adverse outcomes greatly support our hypothesis (Table 3). Consequently, we believe that our findings are still valid. Nonetheless, more studies are needed.

## Conclusion

In this study, we assembled a large cohort of patients with DM and CKD and examined their risk of developing UTI according to their frailty status at the beginning of follow-up. We were able to show that those with a higher severity of frailty, in the forms of increasing FRAIL item counts, carried a rising risk of UTI over 3.51 years of follow-up compared to those without any FRAIL items. For every FRAIL item increase, there was a 11 % increase in the risk of UTI. Based on these findings, it is suggested that the prevention or reduction in frailty severity may exert beneficial influences on the probability of future UTI risk in patients with DM and CKD.

## Data Availability

The raw data for conducting this analysis are unavailable due to the prohibition imposed by the administrative authority of the Ministry of Health and Welfare in Taiwan. The analytical results are available upon reasonable request to the corresponding author.

## Abbreviations

aDSCI: adapted diabetes complications severity index
ANOVA: analysis of variance
CCI: Charlson comorbidity index
CHS: Cardiovascular Health Study
CI: confidence interval
CKD: chronic kidney disease
DKD: diabetic kidney disease
DM: diabetes mellitus
eGFR: estimated glomerular filtration rate
ESRD: end-stage renal disease
HR: hazard ratio
ICD-9-CM: International Classification of Diseases, 9^th^ revision, Clinical Modification
LCDP: Longitudinal Cohort of Diabetes Patients
MNA: mini-nutrition assessment
NHIRD: National Health Insurance Research Database
PPV: positive predictive value
SD: standard deviation
SOF: Study of Osteoporotic Fractures
UTI: urinary tract infection
